# Prevalence and distribution of high-risk human papilloma virus (HPV) types in invasive squamous cell carcinoma of the cervix and in normal women in Andhra Pradesh, India

**DOI:** 10.1186/1471-2334-5-116

**Published:** 2005-12-22

**Authors:** A Pavani Sowjanya, Meenkashi Jain, Usha Rani Poli, S Padma, Manik Das, Keerti V Shah, BN Rao, Radha Rama Devi, Patti E Gravitt, Gayatri Ramakrishna

**Affiliations:** 1 Centre for DNA Fingerprinting and Diagnostics, Hyderabad, A.P, India; 2 Mediciti Rural Hospital, Medchal Mandal, A.P., India; 3 M.N.J. Regional Cancer Hospital, Hyderabad, A.P., India; 4 Johns Hopkins University, Baltimore, USA

## Abstract

**Background:**

Despite the high incidence of cervical cancer reported from India, large scale population based studies on the HPV prevalence and genotype distribution are very few from this region. In view of the clinical trials for HPV vaccine taking place in India, it is of utmost importance to understand the prevalence of HPV genotypes in various geographical regions of India. We investigated the genotype distribution of high-risk HPV types in squamous cell carcinomas and the prevalence of high-risk HPV in cervicovaginal samples in the southern state of Andhra Pradesh (AP), India.

**Methods:**

HPV genotyping was done in cervical cancer specimens (n = 41) obtained from women attending a regional cancer hospital in Hyderabad. HPV-DNA testing was also done in cervicovaginal samples (n = 185) collected from women enrolled in the cervical cancer screening pilot study conducted in the rural community, of Medchal Mandal, twenty kilometers away from Hyderabad.

**Results:**

High-risk HPV types were found in 87.8% (n = 36/41) of the squamous cell carcinomas using a PCR-based line blot assay. Among the HPV positive cancers, the overall type distribution of the major high-risk HPV types was as follows: HPV 16 (66.7%), HPV 18 (19.4%), HPV 33 (5.6%), HPV 35 (5.6%), HPV 45 (5.6%), HPV 52 (2.8%), HPV 58(2.8%), HPV 59(2.8%) and HPV 73 (2.8%). Women participating in the community screening programme provided both a self-collected vaginal swab and a clinician-collected cervical swab for HPV DNA testing. Primary screening for high risk HPV was performed using the Digene Hybrid Capture 2 (hc2) assay. All hc2 positive samples by any one method of collection were further analyzed using the Roche PCR-based line blot for genotype determination. The prevalence of high risk HPV infection in this community-based screening population was 10.3% (19/185) using the clinician-collected and 7.0% (13/185) using the self-collected samples. The overall agreement between self-collected and clinician-collected samples was 92%; however among HPV-positive specimens, the HPV agreement was only moderate (39.1%). The most frequently detected HPV types in the Medchal community are HPV 52 and 16.

**Conclusion:**

Our results suggest that the HPV type distribution in both cervical cancer tissues and in a general screening population from Andhra Pradesh is similar to that reported in India and other parts of the world. We also conclude that an effective vaccine targeting HPV 16 will reduce the cervical cancer burden in AP.

## Background

Cervical cancer is one of the most common malignancies and is the major cause of cancer mortality among Indian women. [1,2]. Cytology screening (e.g., Pap test) is the standard method used for the control of cervical cancer in India, however organized screening programs are rare. Despite the availability of Pap testing even on an opportunistic basis in India, the incidence of invasive cervical cancer remains high, especially in rural India [3-5]. The failure of cytological testing in rural India is likely due to a number of factors which include (a) poor infrastructure, (b) lack of trained health professionals and cytotechnicians, (c) absence of organized community based screening programs and (d) inadequate follow-up of abnormal smears [6].

In the past decade, a strong etiologic association between infection with high-risk HPV types and development of cervical cancer has been established, and vaccines targeting HPV 16 and 18 have been shown to prevent persistent HPV infections in clinical trials [7,8]. Mass immunization with these vaccines has the potential of greatly reducing the cervical cancer incidence in India and elsewhere, though the efficacy of the current vaccine formulations is type-specific, and will only prevent infection with the few types available in the vaccine cocktail. The spectrum of HPV types targeted in current vaccine trials is based largely on the prevalence of HPV types in cancers from the developed world. Because geographical variation in type distributions may exist, knowledge about the distribution of HPV types in cervical cancers and HPV types circulating in the communities in different regions of India would be useful in devising the optimum strategy for vaccination in India [9-11].

Most studies in India have only assessed the prevalence of HPV 16 and 18 in cervical cancer tissues. In spite of the fact that cervical cancer burden in India is high, there are very few large scale studies from India describing either HPV prevalence or type distribution in the general population and in invasive cervical cancer (12,13,14,15). Because of subtle regional cultural differences that exist in various states in India, it is important to describe the distribution of HPV genotypes in cancer cases and community samples from multiple representative populations before these data can be generalized for application in national cancer prevention strategies. The present study reports the HPV type distribution in a rural community (20 Km from Hyderabad city) and invasive cancer samples from women attending the regional cancer centre in Hyderabad.

## Methods

### Collection of cancer specimens

For the cancer study conducted at the MNJ regional cancer hospital (between 2002–2003), women were consecutively recruited at the time of their visit to the cancer clinic. A tissue biopsy was collected from women clinically diagnosed with cervical cancer and who gave consent (n = 45). Following the cervical punch biopsy, a small piece of tissue was sent for histopathology and the rest of the specimen was snap frozen in liquid nitrogen and stored at -70°C. Out of the total 45 women, DNA was extracted from forty-two histopathologically proven cases of squamous cell carcinomas. Histopathology was not available from the remaining 3 participants. The study protocol was approved by all participating institutional bioethical committees.

### Collection of cervical samples in the rural community of Medchal

A community-based sample of women aged 30 years and above was recruited from a single village in Medchal Mandal during the period July–October 2003. These women were participating in a cervical cancer screening pilot study entitled Community Access to Cervical Health, or CATCH. A total of 657 age-eligible women were recruited to participate. Consent was obtained from women participating in the study who had not undergone hysterectomy and were not pregnant. Of the 489 women who were eligible, 190 (38.9%) consented to participate and were enrolled. Of the 190 women, HPV analysis was done for 185 women. Both self-collected vaginal samples and clinician-collected cervical samples were collected using a Digene sampling brush which was placed into Digene STM collection medium. Samples were stored at 4°C for no more than 24 hours. Before aliquoting, the STM tubes are vortexed to dislodge cells and then aliquoted without removing the brush. The aliquoted samples were stored at -80°C.

### Consent from participant women

At the MNJ cancer hospital the attending nurse explained the procedure and written consent obtained. For the Medchal community study, the consent was read aloud to the participants as a group. Each eligible participant was then asked privately if they had any questions regarding study participation and written consent was obtained.

### HPV-DNA testing by Digene Hybrid capture 2

The samples collected in the STM tubes were vortexed to dislodge from the brush and 200 μl aliquots from both the self and clinician collected samples were transported to Centre for DNA Fingerprinting and Diagnostics (CDFD) for HPV testing. Aliquotting was done prior to denaturation of the sample for the hc2 test in order to preserve the integrity of the DNA for future PCR analyses. Each sample was tested for the presence of one or more high risk HPV types using probe B of the Hybrid Capture 2 (hc2) assay (a pool of full length RNA probes specific for HPV 16, 18, 31, 33, 35, 39, 45, 51, 52, 56, 58, 59, and 68) according to the manufacturer's instructions. The hc2 test is based on the hybridization of HPV DNA with RNA specific probes and they hybrid detected by a chemiluminescent assay. The values obtained are recorded as relative light unit (RLU) per positive control (PC). The sample is considered positive for HPV-DNA when the RLU/PC ratio is greater than or equal to one.

### HPV-DNA testing by the Roche PCR Line blot assay

HPV genotyping was performed on all cervical cancer tissues and on cervicovaginal samples which were hc2 positive by either collection method. The PCR and line blot reagents were kindly provided by Roche Molecular Systems, Inc. The cervical punch biopsy (approximately 50 mg) was pulverized using liquid nitrogen, suspended in 1000 μl of digestion buffer (10 mM NaCl, 10 mM Tris-Cl pH 8, 25 mM EDTA, 0.5% SDS and 0.1 mg/ml proteinase K), extracted using standard phenol:chloroform methods [16], and resuspended in 100 ul of TE buffer. DNA was extracted from the cervical samples collected from the women enrolled in CATCH study by taking 70 μl of the STM sample and digesting at 65°C in digestion buffer (20 mM Tris-HCl,1 mM EDTA pH 8.5) containing 200 μg/ml proteinase K and 0.1% of Tween 20 for 1 hour. Following heat inactivation of the proteinase K (95°C for 10 minutes), the DNA sample was precipitated with ethanol and ammonium acetate. The precipitated DNA was suspended in a final volume of 35 μl TE buffer. For PCR, 5 μl of the DNA sample is amplified using a cocktail of the biotinylated PGMY 09/11 and beta-globin primers in a final volume of 100 μl. The PCR product is denatured in 0.4 N NaOH, and subjected to HPV genotyping by the prototype PCR-Line blot assay as described earlier (17,18).

### Statistical analysis

Agreement between self- and clinician-collected samples was calculated using kappa statistics to provide estimates beyond chance agreement. To test for differences in HPV prevalence by age, we calculated Pearson's chi-square. Results were considered statistically significant at p < 0.05. All frequencies, kappa estimates, and chi-square tests were computed using Stata/SE version 9.0 (Stata Corp., College Station, TX).

## Results

### Prevalence and distribution of HPV types in cervical cancer tissues

A total of 42 specimens were tested. The age range of the women was 30 to 65 years with a median age of 55 years. The majority of women attending MNJ for cancer diagnosis and treatment are of low socioeconomic status (average income 1000–1500 Rs per month) based on data from hospital records. However, specific details regarding the socioeconomoic or demographic status were not collected from individual women participating in the invasive cancer study. HPV genotyping of the samples was done using the line blot assay and of the total 42 samples tested, one was negative for beta-globin and was excluded from further analyses. Of the 41 tissues with satisfactory β-globin amplification, 36 (87%) were HPV positive. Among the 36 HPV positive tissues, HPV 16 was the most prevalent type detected (24/36, 66.7%), followed by HPV 18 (7/36 19.4%). Three of the HPV16 positive specimens were co-infected with other HPV genotypes (see Table 1). The remaining tumors were positive for the following HPV types: HPV 33 (2/36, 5.6%), 35 (2/36, 5.6%), 45 (2/36, 5.6%), 52 (1/36, 2.8%), 58 (1/36, 2.8%), 59 (1/36, 2.8%) and 73 (1/36, 2.8%).

**Table 1:** Estimated prevalence of HPV types in squamous cell carcinoma

	Number	Percentage total
Total sample size	41	
HPV Negative	5	12.2
HPV Positives	36	87.8
HPV types		
*Single Infection*		
16	21	58.3
18	6	16.7
33	1	2.8
35	2	5.6
45	2	5.6
59	1	2.8
*Multiple infections*		
16/58/52/33	1	2.8
16/18	1	2.8
16**/73**	1	2.8

### Prevalence and distribution of HPV types in community samples

For the CATCH pilot study a total of 657 age-eligible women were recruited to participate. Of the 489 women who were eligible, 190 (38.9%) consented to participate and were enrolled. Women aged 30–45 were twice as likely to participate (60.4%) relative to the next age group (45–55; 29.8% enrolled). Of the 190 women, HPV analysis was done for 185 women. The median household income reported by the women in the community study was 1500 Rs, which is similar to the general characteristics of the population served at the regional cancer hospital.

Of the 190 women enrolled, 185 had samples collected for HPV testing. HPV genotyping was done in women who tested hc2 positive in either the self- or the clinician-collected sample. Approximately 10.3% (19/185) of clinician-collected cervical samples tested positive for high-risk HPV types by the hc2 assay. The HPV prevalence was similar across age strata (p = 0.72, Table 2). All hc2-positive samples were genotyped using the PGMY PCR line blot analysis. One cervical sample was negative for the β-globin control, leaving a total of 18 of the hc2-positive cervical samples for genotype analysis. By PCR analysis, 13/18 (72%) of the hc2-positive cervical samples were confirmed as high risk HPV positive. The genotype distribution among these women with predominately normal cervical cytology is summarized in Table 3. Multiple genotype infections were detected in 2/13 HPV positive women (15%), resulting in a total of 17 HPV infections among 13 women. In general, HPV 52 was the most common type detected (5/17 infections, 29.4%), followed by HPV 16 (17.6%), HPV 58 (11.7%), and HPV 33 (11.7%). HPVs 62, 59, 39, 18, and 84 were each detected once in the cervical swabs.

**Table 2:** HPV-DNA prevalence in women belonging to different age strata in Medchal community

**Age group**	**Sample size**	HPV Positive samples*	**% HPV positivity**
30–35	48	6	13
36–40	49	5	10
41–45	36	2	6
46 and above	53	6	11
	**186**	**19**	**10**

* This data represents the clinician-collected samples tested by hc2 assay; Pearson's chi-square p = 0.72.

**Table 3:** Comparison of RLU/PC cutoff values for cervicovaginal samples positive by any one method of collection and genotyping of hc2 positive samples using the PCR based line blot assay

**Sample**	**RLU/Co-Clinician collected**	**RLU/Co self Self Collected**	**HPV type Clinician collected**	**HPV type Self collected**
1	**204.27**	**30.8**	16	16
2	**179.86**	*0.3*	52	-
3	**151.97**	*0.7*	52,58,62	58,62
4	**78**	*0.2*	59	-
5	**55**	*0.2*	52	- **
6	**47.33**	**1.6**	39	39
7	**35.74**	**5.6**	52	52
8	**14.58**	**3.9**	-	-
9	**11.35**	**398.8**	18	16,18,31,81
10	**1.86**	*0.2*	16,33,84	-
11	**9.44**	**2.9 (2.95)**	58	58
12	**7.02**	**3.1**	52	52
13	**5.69**	**6.2**	*	*
14	**4.5**	*0.2*	-	-
15	**3.81**	**1.0**	33	-
16	**2.26**	*0.2*	-	-
17	**1.51**	*0.2*	-	-
18	**1.38**	*0.3*	-	-
19	**1.1**	*0.6*	16	-
20	*0.19*	**1.2**	-	-
21	*0.66*	**10.2**	-	62,84
22	*0.97*	**17.4**	-	*
23	*0.19*	**1.2**	-	-

The Rlu/cut-off values are arranged in the descending order for the clinician-collected sample. Values above 1.0 is considered positive for HPV and represented in bold.

– HPV negative; * beta globin negative, ** positive for low risk HPV type

We also tested the paired self-collected swabs from each woman in the community-based study using hc2, followed by genotyping of all HPV positive samples using the PCR line blot assay. Approximately 7.02% (13/185) of the self-collected vaginal swabs were positive for high-risk HPV using the hc2 assay. When tested by PCR, two of the vaginal swabs failed to amplify the β-globin control, leaving a total of 11 hc2-positive self-collected samples available for analysis. Of these, 7 (64%) were confirmed as high-risk HPV positive samples, two of which were positive for multiple HPV genotypes resulting in total of 11 infections. HPV 16 and 52 were each present in 2 of the 11 infections (18%), and HPVs 18, 31, 39, 58, 62, 81, and 84 detected once.

Of the five hc2-positive samples in the clinician-collected set that were not confirmed as positive by PCR, three had RLU/PC value ratios between 1.38 and 2.26, while the remaining two had somewhat higher RLU/PC ratios of 4.5 and 14.38. All four hc2-positive self-collected samples that were PCR negative had RLU/cutoff ratios ranging from 1.17–3.91. One of the self-collected samples although negative by the hc2 assay showed the presence of oncogenic HPV type 52 on line blot analysis.

The overall agreement of the hc2 results between the two sampling methods was good (92.4%, Table 4). However, the percentage agreement among the HPV positive samples was only moderate (39%). A pairwise comparison of the data on the relative light unit/cutoff (RLU/Co) for the 23 samples testing positive for HPV by any one of the collection methods together with the HPV types is shown in Table 3.

**Table 4:** Agreement for HPV positivity in cervicovaginal samples by the two methods of collection

	**Self-Collected samples**		**Total**
**Clinician collected**	Positive	Negative	

Positive	9	10	19
Negative	4	162	166

**Total**	**13**	**172**	**185**

Total agreement: 92.4% (171/185); HPV positivity agreement: 39.1% (9/23); Kappa = 0.52; C.I = 0.38–0.66

## Discussion

The development of HPV vaccines holds tremendous promise for developing countries like India where cervical cancer is the most common malignancy among middle-aged women, particularly in the rural areas [3]. The availability of an HPV vaccine will not only help in curbing the cervical cancer incidence and mortality, but may also bring down the cost burden for cervical cancer screening programmes. Already there is a possibility for an HPV vaccine trial in India under the auspices of the Indian Council of Medical Research [11]. To maximize the cost-effectiveness of the HPV vaccination programmes in India, it is important to understand the distribution of the major HPV types in various geographical regions.

We therefore evaluated the prevalence and distribution of major HPV types in cervical tissues from histologically proven cervical neoplasia (N = 42) collected from women attending a cancer clinic at the regional cancer hospital in Hyderabad, Andhra Pradesh. High risk HPV prevalence was approximately 87.8% in our sample of invasive squamous cell carcinomas. In the present study, low risk HPV types were not detected in the carcinomas. A case-control study undertaken in Chennai, southern India, reported HPV prevalence as high as 99.4% in their invasive cancer samples [12]. Both studies used broad spectrum, consensus PCR methods for the typing of HPV. However, the method used in Chennai amplified an HPV target of ~150 bp, whereas our amplification product using the PGMY 09/11 consensus primers is much larger (450 bp). Therefore, DNA degradation in some of the samples in our study could have led to a false-negative result and an underestimation of the HPV prevalence in cervical cancers from AP State. Similar false negative samples using the degenerate primers MY09/11 (also amplifying 450 bp HPV targets) were found relative to the 150 bp amplicon in a large case-series of cervical tumors conducted by the IARC [19,20].

The most prevalent HPV types found in the invasive cervical cancers in Andhra Pradesh were HPV 16 (66.7%) followed by HPV 18 (19.4%). The distribution of HPV types found in our study is quite similar to a recent large-scale study reported from India and is also consistent with the most common types found in South-East Asia [12-14,21,22]. Therefore, we can confirm that a vaccine targeting HPV 16 could eliminate >50% of the cervical cancer burden in Andhra Pradesh, as well as South India. More comprehensive genotyping of cervical cancer tissues from North, West, and Northeast India will be needed to justify a single national vaccine strategy for the Indian subcontinent.

While the incidence of cervical cancer is high in the state of Andhra Pradesh, with an age adjusted rate of 10.3 per 10,000 women [23] few data are available which describe the prevalence or distribution of major HPV genotypes in the general population. We are conducting a cervical cancer screening evaluation study in rural AP, which includes HPV testing as a primary screening method. The present paper reports the prevalence and distribution of major HPV types in cervicovaginal samples from the women enrolled from a single village in Medchal Mandal as part of the CATCH pilot study. A broad range of genotypes were detected in this community-based sample, including many of the types found in invasive cancers from the same region, suggesting that the population prevalence and spectrum of HPV infection in rural India is similar to that seen elsewhere in the world and India [14,24].

Our HPV prevalence (10.4%) is very similar to two large population-based studies of largely cytologically normal women. Sankarnarayanan, et al. reported 10.3% high risk HPV prevalence as detected using hc2 testing in Osmanabad District in West India [15], and somewhat lower prevalence estimates by hc2 in a separate multicentric study in Mumbai (6.3%), Trivandrum (4.8%), and two cities in Kolkatta (7.8 and 5.2%, respectively) [25]. Franceschi et al report similar high risk prevalence from Dindigul District in South India (9.6%) using consensus primer PCR methods [14]. None of these studies, including ours, found an association of HPV prevalence with age. Our study and that of Sankarnarayanan [26] restricted enrollment to women over age 30 years, which may represent the age-associated plateau found in other reports. However the study from Dindigul District [14] sampled a large number of women under 25 years and found no increase in HPV prevalence among the younger women. The lack of an age association with HPV prevalence in India is yet unexplained.

We evaluated HPV-DNA testing using both clinician- and self-collected samples from each woman. In this pilot study, the initial screening for high risk HPV DNA was done by Hybrid Capture 2 with positive samples tested for HPV genotype using a PCR-based line blot assay. There was a considerable variation in the RLU/PC values for the self and clinician collected samples, however more than half of the discordant samples had viral loads <10 RLU/PC. Similarly, most hc2-positive samples that were not confirmed by PCR had low viral load suggesting sampling error as a potential source of variability when sample viral loads are near the assay sensitivity threshold. Further, it is possible that the discrepancy between hybrid capture positive and PCR negative samples in samples with RLU/CO less than 5 is because of non-specific binding in the hc2 assay leading to false positive results.

Clinician collected samples (10.3%) showed a slightly higher HPV prevalence relative to the self collected samples (7%) in the pilot study. The good overall agreement is reflective of a large number of HPV negative samples; continued evaluation of HPV testing in this community with a larger sample size will be required to determine the relative performance of self- vs. clinician-collected samples for HPV testing. Self-sampling as a means to monitor HPV infection post-vaccination may prove to be a valuable tool in post-immunization surveillance in India.

The most prevalent HPV genotypes in the general population of Medchal is HPV 52 followed by HPV 16, which differs slightly from that of rural community in Chennai where the major types were HPV 16 and 56. However, screening of larger samples size in Medchal will give a better picture on the distribution of the HPV types.

Our efforts are therefore continuing in the characterization of HPV genotypes prevalent in the rural areas of Andhra Pradesh. The successful completion of our ongoing studies will help in (a) understanding the distribution and prevalence of HPV types in Medchal Mandal community of Andhra Pradesh, (b) feasibility of self collection methods for HPV-DNA testing in India as an alternate to clinician based sampling, and (c) comparing the three different screening modalities (Pap test, VIA, and HPV-DNA testing). Furthermore, combining our results with the ongoing IARC study conducted in the Mahabub Nagar district of Andhra Pradesh and the recently completed IARC assisted studies will help in predicting the most cost-effective method of cancer cervix screening programmes in India [27]. A comparative evaluation of such different large scale control studies conducted in India will provide newer insights to formulate better ways to meet the future challenges for cervical cancer prevention in India. Finally, establishment of a well-characterized population with regard to the community prevalence of type-specific HPV infection will provide a valuable baseline for monitoring population effectiveness of an HPV vaccine.

## Conclusion

Our results confirm the earlier studies on the role of high risk HPV infection as a major risk factor development of cancer cervix. Further, the distribution of high risk HPV types in Hyderabad is similar to those reported from a recent study conducted in Southern India. The prevalence of HPV-16 in the cancer samples suggests that effective vaccination against HPV 16 can considerably bring down the cancer burden in the southern states of India.

## Authors' contributions

Keerti Shah and Patti Gravitt were responsible for the overall planning and coordination of the Medchal community based study. Usha Rani Poli, Radha Rama Devi, B.N.Rao helped in providing the invasive cancer specimens for the study. Pavani and Padma were involved in the cancer cervix sample collection. Pavani was responsible for HPV-DNA testing on all the samples using line blot and Hybrid capture-II assay. Meenakshi Jain collected all the cervicovaginal samples from women enrolled in the CATCH programme. Manik Das was responsible for biospecimen processing and inventory. Patti Gravitt provided all the expertise involved with the HPV Line blot assay system. Gayatri Ramakrishna was involved in the coordination and setting up of the HPV-DNA testing system at CDFD and together with Patti Gravitt compiled and finalized the manuscript.

## Declaration of Competing interests

The author(s) declare that they have no competing interests. We have received reagents from Roche Molecular Systems. These are the same reagents used in this manuscript.

## Pre-publication history

The pre-publication history for this paper can be accessed here:


